# Influence of Various Bottom DBR Designs on the Thermal Properties of Blue Semiconductor-Metal Subwavelength-Grating VCSELs

**DOI:** 10.3390/ma12193235

**Published:** 2019-10-02

**Authors:** Robert P. Sarzała, Łukasz Piskorski, Tomasz Czyszanowski, Maciej Dems

**Affiliations:** Institute of Physics, Lodz University of Technology, 90-924 Lodz, Poland; robert.sarzala@p.lodz.pl (R.P.S.); tomasz.czyszanowski@p.lodz.pl (T.C.); maciej.dems@p.lodz.pl (M.D.)

**Keywords:** vertical-cavity surface-emitting lasers, subwavelength gratings, numerical simulations

## Abstract

In this paper, we consider several designs for nitride-based vertical-cavity surface-emitting lasers (VCSELs) with a top semiconductor-metal subwavelength grating (SMSG) as the facet mirror. The constructions of the bottom distributed Bragg reflectors (DBRs) used in the VCSEL designs were inspired by devices demonstrated recently by several research groups. A multiparameter numerical analysis was performed, based on self-consistent thermal and electrical simulations. The results show that, in the case of small aperture VCSEL designs, dielectric-based DBRs with metallic or GaN channels enable equally efficient heat dissipation to designs with monolithically integrated DBRs. In the case of broad aperture designs enabled by SMSGs, monolithically integrated DBRs provide much more efficient heat dissipation in comparison to all other considered designs.

## 1. Introduction

Gallium nitride-based vertical-cavity surface-emitting lasers (VCSELs) are currently under development, with potential for use as light sources in optical communication, optical storage, laser printers, and biosensors [1,2,3,4]. GaN-based VCSELs could also provide the missing green element in designs for white light emitting pico-projectors, laser headlamps, and high power displays [1,2,3,4,5]. One of the main issues hindering the commercial production of nitride-based VCSELs is the fabrication of distributed Bragg reflectors (DBRs) with satisfactory optical, thermal and electrical properties. It is well known that DBRs with high power reflectance improve the quality factor of the optical cavity, which in turn reduces the threshold current of the device. High power reflectance can be achieved by using a large number of DBR pairs and/or by using pairs of layers with large refractive index contrast. However, high power reflectance is not the only factor determining the choice of DBR materials. High performance DBRs should also be characterized by high electrical conductivity, enabling current injection directly into the active region, high thermal conductivity to improve heat dissipation from the laser, the possibility of monolithic integration with the optical cavity and low lattice mismatch between the different materials used in the laser structure.

The nitride-based DBRs reported thus far in the literature show poor electrical conductivity for both p- and n-type materials. In the case of AlGaN/GaN DBRs, the acceptable mismatch between lattice constants in the layers results in low refractive index contrast and hence many DBR periods are necessary to achieve total reflectivity. Increasing the refractive index contrast by raising the mole fraction of aluminum in AlGaN leads to defects in the crystal lattice and consequently to lower both electrical conductivity and laser efficiency [6]. The problem of stress induced in nitride-based DBRs has been partially solved by placing AlN/GaN superlattice (SL) layers between the DBR pairs. VCSELs with this type of DBR were reported by Lu et al. [7]. However, the large number of thin layers in DBR increases its thermal resistance. Cosendey et al. [8] proposed a DBR composed of lattice-matched AlIn${}_{0.2}$N/GaN, characterized by the refractive index contrast Δn/n equal to 8%, which is two times higher than the one for Al${}_{0.2}$GaN/GaN DBRs [9]. On the other hand, the current injection through the AlInN/GaN DBR is very poor due to large conduction band offsets and strong polarization fields, which leads to a significant voltage drop at each interface [10]. Moreover, the effective thermal resistivity of AlInN/GaN DBR is high, due to low thermal conductivity of AlInN lattice-matched to GaN [11]. Very high value for the refractive index contrast can be obtained with the use of nanoporous GaN DBRs. With the use of data presented in [12], we calculated Δn/n=33% for the above DBRs.

Dielectric DBRs offer an alternative to nitride-based DBRs. However, they are not free from drawbacks. On the one hand, they have high-contrast refractive index and on the other they are insulators with very low thermal conductivity. Dielectric DBRs require an intracavity injection scheme, which can be achieved using a tunnel junction (TJ) or indium tin oxide (ITO) layer. The fabrication of VCSELs composed of two dielectric mirrors [13,14] requires substrate lift-off from the optical cavity, polishing, wafer fusion of both DBRs and etching. These complex processes involve multiple technological steps, hindering commercial production.

In the past, high-contrast gratings (HCGs) have been investigated as a possible alternative to DBRs, enabling the vertical dimension of VCSEL to be reduced, emitted wavelength tuning and polarization stability [15]. However, HCGs require a combination of high refractive index materials and low refractive index materials, which are typically dielectrics or air-gap regions. When incorporated into an electrically driven VCSEL, these materials cause problems for current injection, reduce heat dissipation and are susceptible to vibrational instability [16].

Recently, we proposed a monolithic HCG (MHCG) providing total optical power reflectance for refractive indices of MHCGs larger than 1.75 [17]. Our numerical findings were confirmed by experimental characterization of the power reflectance spectrum [18] of a stand-alone MHCG fabricated from GaAs and designed for a wavelength of 980 nm. In [19], Kim et al. reported first MHCG-cavity polariton laser and later we showed the first electrically-injected MHCG VCSEL with peak emission wavelengths of 980 nm and continuous wave lasing up to 75 ${}^{\circ }$C [20].

**Figure 1:**
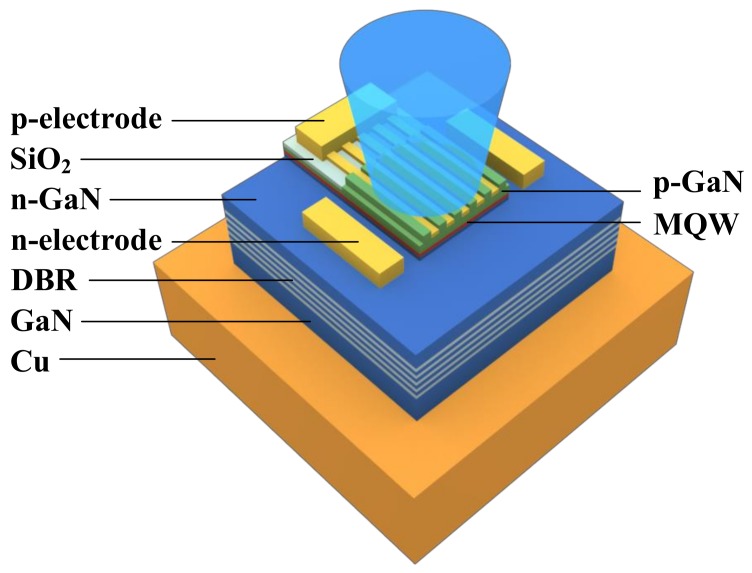
Schematics of the SMSG VCSEL configuration, consisting of a top SMSG with p-electrode. The multi-quantum well (MQW) active region is embedded in the GaN cavity. The n-electrodes are implemented on an n-type GaN above the bottom DBR.

We also proposed an original approach to current injection based on the use of a semiconductor-metal subwavelength grating (SMSG) as the top mirror of a VCSEL [21]. This solution enables a new approach to designing the top part of a VCSEL. A monolithic grating with metal stripes positioned between the semiconductor stripes (Figure 1) provides nearly total optical power reflection and is less susceptible to mechanical instability with respect to HCG. Fabrication of the mirror does not therefore require the combination of semiconductor layers with different lattice constants. The SMSG ensures direct, uniform current injection into the active region of the VCSEL, providing a tenfold increase in emitted power from broad aperture devices [22] in comparison to contemporary devices [7,13,14,23]. The SMSG eliminates the need for a top dielectric DBR and intracavity injection scheme, making it possible to fabricate a monolithic VCSEL without a tunnel junction or ITO layer, and without dielectric or proton-implanted current confinement. However, because the SMSG facilitates injection into broad area apertures, high levels of heat can be generated, requiring more efficient heat management. Heat generated in the active region of a VCSEL must be transported through the bottom DBR towards the heatsink. The bottom DBR thus becomes a crucial element for efficient heat dissipation. In the research presented in this paper, we therefore conducted an operational analysis of SMSG VCSELs with different designs of bottom DBR, to evaluate their thermal properties.

In Table 1, various DBRs fabricated in different material systems for a number of wave regimes are compared in terms of their thermal properties. An arsenide-based DBR composed of binary AlAs/GaAs material fabricated for a VCSEL emitting at 850 nm serves as the reference for three different DBR designs used in nitride-based VCSELs emitting at 410 nm. Table 1 also compares exemplary properties of DBRs used in monolithic VCSEL designs emitting at 650 nm and 1300 nm. Analysis of the parameters in Table 1 reveals that nitride-based VCSELs are expected to suffer similar difficulties to those associated with monolithically integrated phosphide-based VCSELs. The SiO${}_{2}$/Ta${}_{2}$O${}_{5}$ DBRs used in nitride-based constructions provide very high power reflectance, but the fact they are insulators makes current injection into the active region very challenging. DBRs made with a binary AlN/GaN system could potentially provide very attractive thermal properties. However, due to the lattice constant mismatch between the GaN and AlN, they cannot be implemented in real-world devices. The insertion of a small-period superlattice layer between every few DBR pairs [7] decreases strain, which reduces the density of crystal defects in GaN/AlN DBRs. On the other hand, the superlattice composed of a large number of nanometer thick layers reduces the thermal conductivity of DBR, therefore the thickness of the superlattice must be optimized taking into account the beneficial influence on stress reduction and the negative effect on thermal resistance.

An alternative approach is the use of lattice-matched AlInN/GaN DBRs [23], which can provide acceptable thermal properties. Their refractive index contrast is rather low compared to other DBR designs; however, short-wavelength emission requires a total thickness of no more than 4 μm, which is smaller in comparison to longer wavelength designs, suggesting more efficient heat dissipation. Dielectric DBRs are characterized by high thermal resistance, which significantly affects the performance of VCSELs with such mirrors. In [14], metal channels in the form of rings were proposed to allow heat flux to bypass the DBR and hence more efficient transfer of heat to the heat-sink.

## 2. Nitride VCSEL Structures and Numerical Model

**Figure 2:**
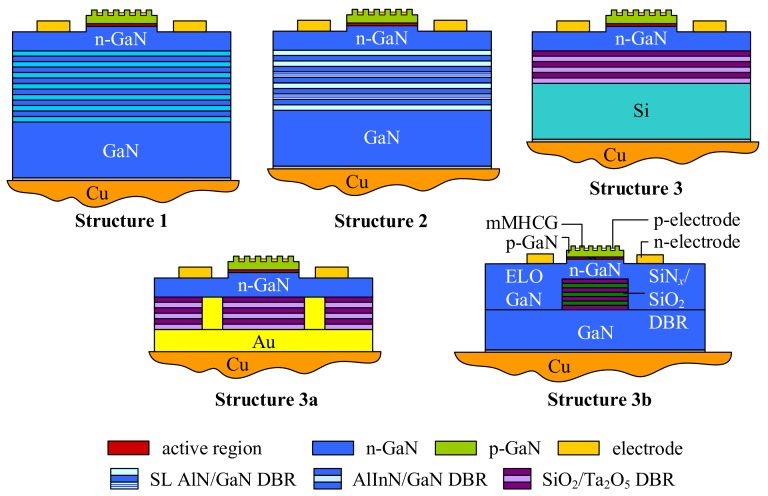
Schematics of considered VCSEL designs. The designs have the same top mirrors and active regions, but differ with respect to the composition and design of the bottom DBR: Structure 1, AlInN/GaN; Structure 2, AlN/GaN with SL layers; Structure 3, a uniform SiO${}_{2}$/Ta${}_{2}$O${}_{5}$ deposited on silicon; Structure 3a, SiO${}_{2}$/Ta${}_{2}$O${}_{5}$ deposited on gold with gold channels that serve as the bottom electrical contact; amd Structure 3b, SiO${}_{2}$/SiN${}_{x}$ embedded in epitaxial, laterally-overgrown (ELOG) GaN.

We analyzed and compared several designs for nitride-based VCSELs with semiconductor-metal subwavelength gratings in terms of thermal and electrical properties. All designs have similar cavity constructions, but their bottom DBRs have various compositions. The schematics of the simulated devices are shown in Figure 2. The VCSEL constructions are designed for a wavelength of 414 nm. The square-shaped semiconductor-metal subwavelength grating plays the role of a facet mirror as well as enabling current injection directly into the active region through silver stripes deposited between undoped GaN stripes (∼150 nm thick). The GaN-Ag SMSG period is 370 nm and the period duty cycle is close to 0.5 [22]. Current is injected through the silver stripes to the p-type GaN spacer, which is the sole p-type region in the design. Lateral optical confinement is induced by the last three stripes of the SMSG, which have a different period to the central stripes. The mechanism of current injection, together with the thermal stability and emitted power of the GaN-Ag SMSG VCSEL under continuous-wave operation, were described in detail in [22].

The active region is composed of five 3-nm thick In${}_{0.09}$GaN quantum wells, separated by 10-nm GaN barriers. Electron leakage from the active region is reduced by a 20-nm thick Al${}_{0.20}$GaN blocking layer. The thickness of the p-GaN spacer between the SMSG and the active region is 100 nm. The minimum thickness of the n-GaN spacer is 117 nm. We vary the total thickness of the cavity from 2 λ to 10 λ where λ is the resonant wavelength in the cavity. This corresponds to variations in the thickness of the GaN layer positioned below the active region. Several bottom DBR compositions are considered. The details of mMHCG (metallic MHCG) VCSELs considered in this work are presented in Table 2. The structures are mounted on a copper heat sink using 2-μm thick PbSn solder.

We use a self-consistent model, combining thermal and electrical submodels to simulate VCSEL operation [29,30]. The temperature distribution in the continuous-wave (CW) operation regime is determined by the following heat transfer equation:

(1) ∇(k(x,y,z)·∇T(x,y,z))=−g(x,y,z),

where *k* is the thermal conductivity of the medium, *T* is temperature and *g* is the volumetric power density of the heat sources. The volumetric power density of the heat sources is described by the following equations:

(2) $$\begin{array}{cc} \gamma & =j\cdot \nabla V, \end{array}$$

(3) $$\begin{array}{cc} j & =\mathrm{diag}(\sigma_{x},\sigma_{y},\sigma_{z})\nabla V, \end{array}$$

where $\mathrm{diag}(\sigma_{x},\sigma_{y},\sigma_{z})$ is a diagonal matrix with the diagonal coordinates of electrical conductivity tensor σ and *V* is the electric potential. Equations (1) and (2) are solved using the Finite Element Method. In the VCSEL structure, the most intensive heat extraction is observed through the bottom Cu-heat sink [31]. Therefore, we assume that the other sides of the laser are thermally isolated and the bottom of the copper heat-sink is kept at a constant temperature of 300 K.

The material parameters used in the calculations, in particular the thermal and electrical conductivities for selected temperatures, are given in [22]. Their dependencies on temperature, level and type of dopant, layer thickness, etc. can be found in [27]. These dependencies have been used previously in the analysis of nitride-based VCSELs with an ITO contact layer and tunnel junctions [32,33,34].

## 3. Results

In this section, we consider Structures 1–3. Modifications of Structure 3 (Structures 3a and 3b) are addressed in Section 3.1. All structures are described in detail in Section 2. The constructions differed with respect to the bottom DBR design. All calculations were performed assuming an average density of the current in the active region of 5.5 kA·cm${}^{-2}$, which is the approximate threshold value of a typical nitride-based VCSEL with an electrical aperture ($W_{\mathrm{active}}$) of 8 μm [14]. The first analyzed VCSEL structure has an AlInN/GaN bottom DBR (Structure 1). Due to the matching between the lattice constants of AlInN with 18% indium content and GaN [8,35,36], such mirrors are expected to show superior mechanical stability in comparison to the other two constructions. The indium content reduces the refractive index contrast and power reflectance of 99.9% can be obtained with 47 DBR pairs [1,8]. The large number of DBR layers, together with the low thermal conductivity of AlIn${}_{0.17}$N at 4.87 W/(m·K) [11], contributes to the significant thermal resistance of the device.

**Figure 3:**
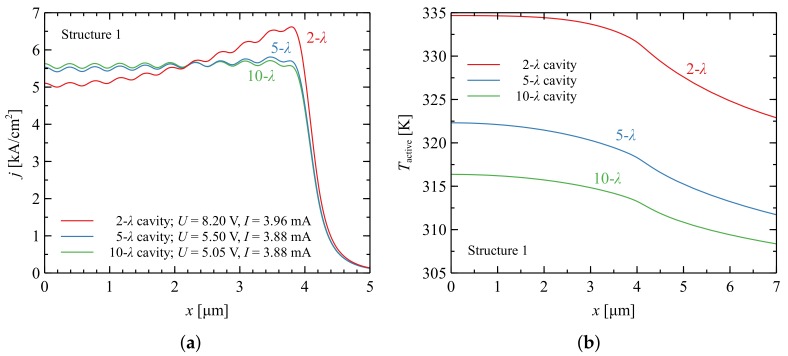
Current density (**a**) and temperature (**b**) distributions within the active region of a nitride-based VCSEL with AlInN/GaN DBR (Structure 1). The active region size is 8 × 8 μm${}^{2}$. Different curves correspond to various thicknesses of the cavities from 2 λ to 10 λ.

Figure 3a shows the distribution of the current density injected into the active region of the laser and the corresponding temperature distributions within the active layer. Increasing the cavity thickness leads to more efficient heat dissipation, due to more efficient lateral heat spreading in the bulky uniform material of the cavity. This mechanism is more noticeable in the case of AlInN/GaN DBRs, which have low efficiency in terms of thermal conductivity. In the case of short cavities, the electrical resistance of the devices is increased due to the narrow lateral current path in n-type GaN. To achieve the expected current density in the active region requires higher voltage and therefore greater electrical power supplied to the VCSEL. This mechanism favors nonuniform current injection and the accumulation of current at the edges of the active region (Figure 6b in [22]), which induces lateral maxima in the temperature distribution. Current density oscillations shown in Figure 3a are the result of multiple separated contacts and poor electrical conductivity of p-GaN layer.

To explore the possibility of improving thermal management, a very similar structure was considered to that proposed in [7], in which the bottom DBR is composed of AlN and GaN quarter-wavelength pairs separated by a short-period superlattice (Structure 2). Since the structures were similar, the current density distributions within the active region were very close to those presented in Figure 3a. However, replacement of relatively thick and of low thermal conductivity AlInN/GaN DBR with SL AlN/GaN DBRs led to temperature reduction in the laser.

**Figure 4:**
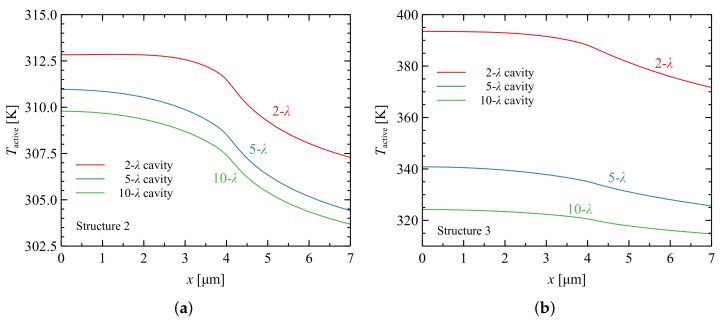
Temperature distributions within the active region of a nitride-based VCSEL with DBRs composed of: 35 pairs of SL AlN/GaN (Structure 2) (**a**); and 11 pairs of SiO${}_{2}$/Ta${}_{2}$O${}_{5}$ (Structure 3) (**b**). The active region size is 8 × 8 μm${}^{2}$. Consecutive curves correspond to various thicknesses of the cavities from 2 λ to 10 λ.

Comparison of Figure 3b and Figure 4a reveals a 40% reduction in temperature in the active region for constructions with a cavity thickness of 10 λ. The temperature reduction in the case of thinner cavities was even larger.

**Figure 5:**
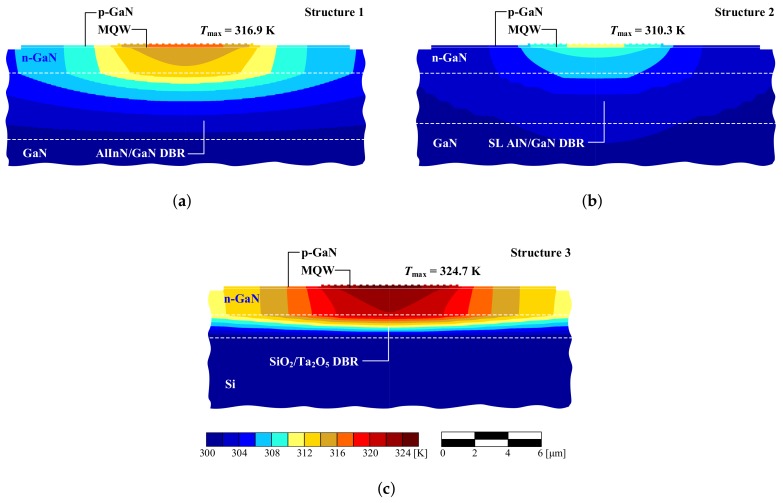
Temperature distribution in 8 × 8 μm${}^{2}$ mMHCG VCSEL with 10-λ cavity and: (**a**) an AlInN/GaN DBR (Structure 1); (**b**) an AlN/GaN DBR with SL layers (Structure 2); and (**c**) a SiO${}_{2}$/Ta${}_{2}$O${}_{5}$ DBR (Structure 3) in the plane perpendicular to the epitaxial layers.

Calculations for an analogous laser with a SiO${}_{2}$/Ta${}_{2}$O${}_{5}$ DBR (Structure 3) were also performed. These materials are characterized by poor thermal conductivity, which results in elevated temperatures during laser operation. The temperature distribution within the active region of the VCSEL with a SiO${}_{2}$/Ta${}_{2}$O${}_{5}$ DBR is shown in Figure 4b. As can be seen, in the case of a 10-λ cavity the temperature in the active region increased more than twofold in comparison to the AlN/GaN DBR with SL (Structure 2), while in the case of a 2-λ cavity the temperature increased nearly eightfold. With a thin cavity, the temperature increase was larger (especially when the thermal resistance of the DBR was higher). Increasing the thickness of the cavity improved lateral heat spread, enabling more efficient heat flow through the DBR mirror towards the heat-sink. A thick GaN cavity acts in a similar manner to a heat-spreader [22], which according to Fourier’s law reduces the temperature inside the device by widening the heat flux. Figure 5 illustrates the temperature distributions in the plane perpendicular to the epitaxial layers in Structures 1–3.

The low effective thermal conductivity of AlInN/GaN DBRs (Figure 5a, Structure 1) deteriorates the vertical heat flow, which is visible as a high temperature gradient. Lateral heat flow therefore increases, as can be seen from the increased temperature in lateral regions of the VCSEL. Figure 5b shows efficient heat management in SL AlN/GaN DBRs (Structure 2), as evidenced by the relatively low temperature increase in the cavity and low temperature gradients. Figure 5c reveals more intense heat accumulation in the cavity, where the dielectric DBR (Structure 3) blocks the vertical heat flux, contributing to a significant rise in temperature within the device.

### 3.1. VCSELs with Dielectric DBR

**Figure 6:**
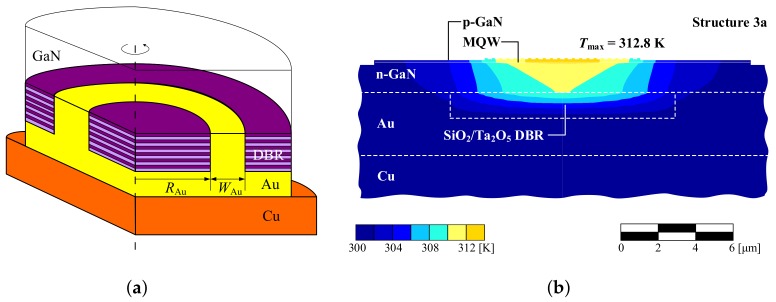
Sketch of the DBR with gold channel in Structure 3a (**a**), and temperature distribution in 8 × 8 μm${}^{2}$ mMHCG VCSEL with 10-λ cavity and SiO${}_{2}$/Ta${}_{2}$O${}_{5}$ DBR and gold channels (Structure 3a) in the plane perpendicular to the epitaxial layers (**b**). The Au ring inner radius ($R_{\mathrm{Au}}$) is 6 μm and its width ($W_{\mathrm{Au}}$) is 5 μm.

**Figure 7:**
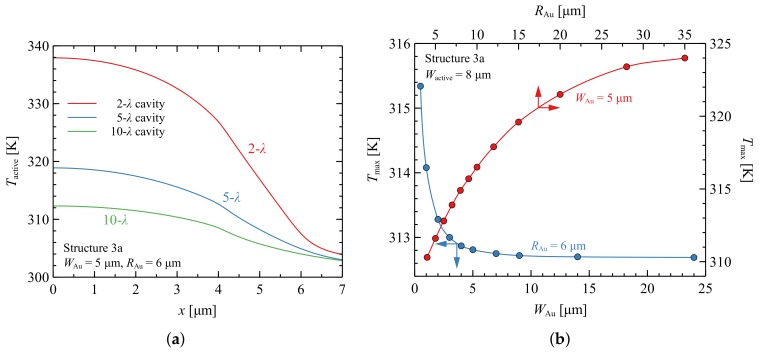
(**a**) Temperature distributions within the active region of the nitride-based VCSEL with SiO${}_{2}$/Ta${}_{2}$O${}_{5}$ DBR and gold channel (Structure 3a). The active region size is 8 × 8 μm${}^{2}$. Different curves correspond to various lengths of the cavities from 2 to 10 λ. (**b**) Maximal temperature in the active region as a function of the channel width ($W_{\mathrm{Au}}$) for $R_{\mathrm{Au}}=6$μm (blue line) and as a function of Au ring inner radius ($R_{\mathrm{Au}}$) for $W_{\mathrm{Au}}=5$μm (red line).

Thermal management in VCSELs with SiO${}_{2}$/Ta${}_{2}$O${}_{5}$ DBRs can be significantly improved by introducing channels between the cavity and heat-sink filled with Au (Structure 3a) [37]. These channels, in the shape of a hollow ring filled with gold, are characterized by a ring inner diameter $2R_{\mathrm{Au}}$ and channel width $W_{\mathrm{Au}}$, as depicted in Figure 6a. Figure 6b presents the temperature distribution in Structure 3a with $R_{\mathrm{Au}}=6$μm and $W_{\mathrm{Au}}=5$μm. In this design, heat flow bypasses the DBRs with high thermal resistance, contributing to significant temperature reduction in the active region of the VCSEL (Figure 7b) in comparison to the design without gold channels (see Figure 5c, Structure 3).

Gold channels improve heat extraction from the active region, reducing the total thermal resistance of the device to a level comparable to that of VCSEL with SL AlN/GaN DBRs (Structure 2). These conclusions are confirmed in Figure 7a, which shows temperature distributions in the case of a 10-λ cavity. With shorter cavities, e.g., 2-λ, the temperature increase in the centre of the active region is three times greater in comparison to that in the case of a VCSEL with a SL AlN/GaN DBR (Structure 2), due to the reduced possibility of lateral heat spreading. However, the temperatures for 10-λ cavities in the two designs are comparable, which indicates that heat-spreading is the crucial mechanism for thermal management in the case of thick cavities.

An important question concerns the optimal parameters for the gold channels. Figure 7b illustrates the influence of gold channel width and radius on maximal temperature in the active region. In the figure, the blue curve represents the maximal temperature dependence in the active region as a function of the width of the ring ($W_{\mathrm{Au}}$), while $R_{\mathrm{Au}}$ remains unchanged. The red curve shows changes in the inner radius (i.e., the position) of the ring, without changing its width. The results show that increasing the channel width $W_{\mathrm{Au}}$ above 5 μm no longer leads to a significant reduction in the temperature of the active region. In the considered design for a VCSEL with an SMSG of 8 × 8 μm${}^{2}$, the channel should be placed no closer than 6 μm from the centre of the structure. If the channel is closer, this leads to severe interaction between the gold and the optical mode, contributing to significant optical losses.

**Figure 8:**
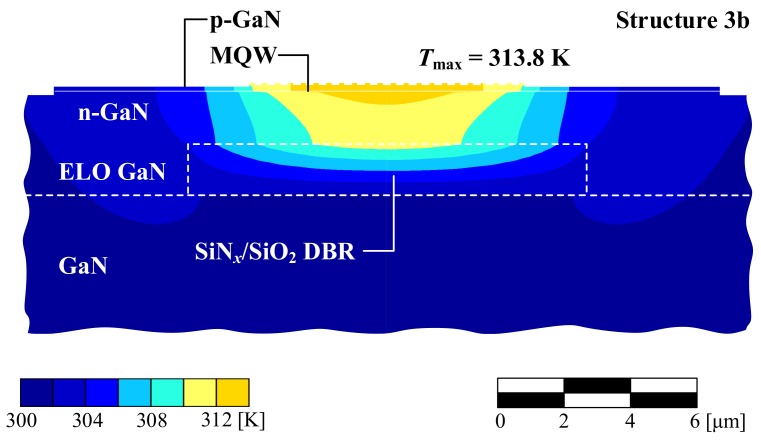
The temperature distribution within 8 × 8 μm${}^{2}$ mMHCG VCSEL with 10-λ cavity and a SiO${}_{2}$/SiN${}_{x}$ DBR embedded in ELOG GaN (Structure 3b) in the plane perpendicular to the epitaxial layers.

A similar concept enabling heat to bypass the DBR was proposed by SONY [38], in which a SiN${}_{x}$/SiO${}_{2}$ DBR is embedded in a GaN substrate (Structure 3b), eliminating the absorption in the gold channels discussed in the case of the previous structure. The DBR is overgrown by GaN, creating a bulk layer above the DBR on which the epitaxial layers of the VCSEL are deposited. Such a solution ensures efficient heat dissipation from the active region, resulting in a comparable temperature increase (see Figure 8) with respect to the VCSEL with gold channels and the VCSEL with an SL AlN/GaN DBR.

There are a number of possible approaches to the fabrication of bottom DBRs in the case of small aperture VCSELs, offering efficient heat extraction from the laser. In terms of the technological effort required, fabricating a VCSEL with a SiO${}_{2}$/Ta${}_{2}$O${}_{5}$ DBR and gold channels presents the greatest challenge. A monolithically integrated VCSEL with an SL AlN/GaN DBR can be fabricated in a single epitaxial growth process. However, growing a high quality DBR with a large number of periods is challenging. A VCSEL with a SiN${}_{x}$/SiO${}_{2}$ DBR, although technologically more complex, offers the significant advantage of requiring a smaller number of DBR pairs to achieve a high level of power reflectance.

### 3.2. Broad Area VCSELs

**Figure 9:**
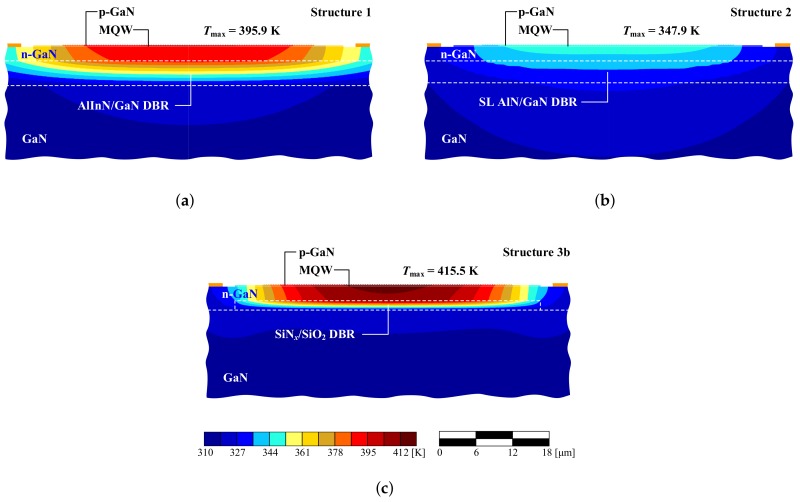
The temperature distribution within 40 × 40 μm${}^{2}$ mMHCG VCSEL with 15-λ cavity and: (**a**) an AlInN/GaN DBR (Structure 1); (**b**) an AlN/GaN DBR with SL layers (Structure 2); and (**c**) a SiO${}_{2}$/SiN${}_{x}$ DBR embedded in ELOG GaN (Structure 3b), in the plane perpendicular to the epitaxial layers.

SMSG VCSELs enable vertical current injection into very large active regions, up to 40 × 40 μm${}^{2}$ [22]. This is significantly larger than the active regions discussed in the previous section, and larger than those typically used in nitride-based VCSELs [7,13,14,23]. The threshold currents of such broad area VCSELs generate significantly more heat, which rises in proportional to the area of active region. In broad area VCSELs, monolithically integrated DBRs of low thermal resistivity enable efficient heat extraction from the active region. Figure 9a,b shows temperature maps in the case of 40 × 40 m${}^{2}$ mMHCG VCSELs with 15-λ cavity and with an AlInN/GaN DBR (Structure 1) and an SL AlN/GaN DBR (Structure 2). Structure 2 provides the lowest temperature increase, at almost half that for the laser with AlInN/GaN mirrors (Structure 1), inside the laser under comparable electric power condition. Dielectric DBRs of low thermal efficiency contribute to a strong increase in temperature in the active region, which makes it impossible to reach the threshold of laser operation in the broad area VCSEL.

The modified Structures 3a and 3b enable heat flux to bypass the dielectric DBRs. For example, Figure 9c illustrates temperature distribution in Structure 3b, with high temperature accumulation in the proximity of the active region and lateral heat-flow according to the temperature gradient. Although heat escapes laterally through GaN regions, the maximal temperature increase in the structure is larger than in Structure 1 and twice larger than in Structure 2. The corresponding density of a current, compared to our results presented in [22], is still high enough to allow assuming that obtaining the lasing threshold is possible.

## 4. Conclusions

This paper presents an extensive thermal analysis of new designs for a nitride-based semiconductor-metal subwavelength grating (SMSG) vertical-cavity surface-emitting laser (VCSEL) with various constructions of bottom distributed Bragg reflectors (DBRs). We considered bottom DBRs composed of AlInN/GaN (Structure 1), AlN/GaN with superlattice (SL) (Structure 2), SiO${}_{2}$/Ta${}_{2}$O${}_{5}$ (Structure 3) and SiO${}_{2}$/Ta${}_{2}$O${}_{5}$ embedded in a gold contact ring (Structure 3a) or in GaN (Structure 3b). Each of these constructions was characterized by different thermal resistance (Table 3) and a different mechanism of heat dissipation. Fully dielectric mirrors showed the highest thermal resistance, while the lowest was observed in the case of the DBR with AlN/GaN layers and SL. Our results suggest that dielectric DBRs integrated with gold channels enable equally efficient heat extraction as AlN/GaN DBRs with SL. An additional advantage of SiO${}_{2}$/Ta${}_{2}$O${}_{5}$ DBRs embedded in a gold contact ring is the much higher power reflectance of the mirror in comparison to AlN/GaN DBRs, since the number of DBR periods in this configuration is limited to the lattice mismatch between AlN and GaN. The heat dissipation efficiency of dielectric mirrors with gold channels is particularly high for lasers with a thick resonator. A similar effect was also observed in the case of the design with a SiN${}_{x}$/SiO${}_{2}$ dielectric mirror embedded in epitaxial, laterally overgrown GaN. Our analysis concerned a VCSEL with a standard aperture size of 8 × 8 μm${}^{2}$. The proposed design for the SMSG VCSEL, however, enables fabrication of devices with much wider apertures, as large as 40 × 40 μm${}^{2}$. In this case, we showed that designs based on dielectric mirrors, even those equipped with heat removal elements (gold channels, DBR overgrown with GaN), introduce large thermal resistance due to the long lateral heat extraction path. The optimal solution in the case of broad area VCSELs would be a mirror composed of high thermal conductivity materials, for example AlN/GaN with SL.

## Figures and Tables

**Table 1:** Comparison of the thermal parameters of DBRs used in nitride-based 410-nm VCSELs and DBRs in VCSELs designed for longer wavelengths. All constructions provide 99.9% power reflectance. λ, wavelength; Δn/n, refractive index contrast; $h_{\mathrm{tot}}$, total thickness; $k_{z,\mathrm{eff}}$, effective thermal conductivity in *z*-direction; RTR, relative thermal resistance. Values of $k_{z,\mathrm{eff}}$ were calculated with the use of formula found in [24] and thermal conductivities for SiO${}_{2}$ [25], Ta${}_{2}$O${}_{5}$ [26], nitrides [27], arsenides [28], and phosphides [28]. Values of RTR were calculated with the use of thermal resistances calculated as a ratio of $h_{\mathrm{tot}}$ to $k_{z,\mathrm{eff}}$.

λ [nm]	Material	Δn/n [%]	No. of Pairs	$h_{\mathrm{tot}}$ [μm]	$k_{z,\mathrm{eff}}$ [W/(m·K)]	RTR
410	SiO${}_{2}$/Ta${}_{2}$O${}_{5}$	33.3	10	1.1	0.70	34
410	AlN/GaN	11.3	35	3.1	118.45	0.6
410	AlInN/GaN	8.5	46	4.0	8.64	10
650	AlGaAs/AlGaAs	11.6	33	3.3	19.55	3.6
850	AlAs/GaAs	17.5	22	2.9	62.25	1
1300	InGaAsP/InP	5.3	77	15.2	14.09	23

**Table 2:** Details of DBR designs used in VCSEL Structures 1–3 (Figure 2): number of DBR pairs, materials, layer thickness *h*, corresponding refractive indices *n*, thermal conductivities *k*, and relative thermal resistances RTR at 300 K.

Structure	No. of Pairs	Material	*h* [nm]	*n* [-]	*k* [W/(m·K)]	RTR
1	46	AlIn${}_{0.18}$N GaN	44.9 41.3	2.306 2.506	4.87 72.4	17
2	35	AlN GaN	46.6 41.3	2.221 2.506	270 72.4	1
3a	11	SiO${}_{2}$ Ta${}_{2}$O${}_{5}$	69.5 46.4	1.489 2.331	1.38 0.4	70
3b	12	SiO${}_{2}$ SiN${}_{x}$	69.5 52.5	1.489 1.969	1.38 2.00	35

**Table 3:** Thermal resistances $R_{\mathrm{th}}$ and maximum temperatures $T_{\max}$ for the modelled structures.

Structure	Active Area [μm${}^{2}$]	$R_{\mathrm{th}}$ [K/W]	$T_{\max}$ [K]
1 (Figure 5a)	8 × 8	3480	316.9
2 (Figure 5b)	8 × 8	2140	310.3
3 (Figure 5c)	8 × 8	5279	324.7
3a (Figure 6b)	8 × 8	2613	312.8
3b (Figure 8)	8 × 8	2832	313.8
1 (Figure 9a)	40 × 40	748	395.9
2 (Figure 9b)	40 × 40	359	347.9
3b (Figure 9c)	40 × 40	901	415.5
